# Synthesis of hypergrafted poly[4-(N,N-diphenylamino)methylstyrene] through tandem anionic-radical polymerization of radical-inimer

**DOI:** 10.1080/15685551.2017.1365577

**Published:** 2017-08-30

**Authors:** Minglu Huang, Jianmin Lu, Bingyong Han, Xianhong Zhang, Wantai Yang

**Affiliations:** ^a^ State Key Laboratory of Chemical Resource Engineering, Beijing University of Chemical Technology, Beijing, P.R. China

**Keywords:** Self-condensing vinyl polymerization, hypergrafted polymer, poly(4-(N, N-diphenylamino)methylstyrene)

## Abstract

In this paper, we present a tandem anionic-radical approach for synthesizing hypergrafted polymers. We prepared 4-(N,N-diphenylamino)methylstyrene (DPAMS) as a new radical-based inimer. Linear PDPAMS was prepared through anionic polymerization. Hypergrafted PDPAMS was synthesized through the self-condensing vinyl polymerization of DPAMS with linear PDPAMS. The linear backbone of PDPAMS, which incorporated latent radical initiating sites, served as a ‘hyperlinker’ to link hyperbranched side chains. The molecular weights of hypergrafted polymers increased as the length of the linear backbone chain increased. The hypergrafted structure of the resulting polymer was confirmed using a conventional gel permeation chromatograph apparatus equipped with a multiangle light scattering detector, nuclear magnetic resonance, differential scanning calorimetry, and thermogravimetric analysis. This strategy can be applied to synthesize other complex architectures based on hyperbranched polymers by changing the structure of a polymer backbone through anionic polymerization.

## 1. Introduction

Polymers with hyperbranched structures have gained increasing research interest for the preparation of polymer topologies with unique properties; potential areas of application include supramolecular assembly, drug delivery, and catalysis, etc. [1–9]. The emergence of innovative synthesis strategies makes it possible to synthesize specially shaped polymers with hyperbranched architectures. The initiator-monomer (inimer) strategy is one promising example of a strategy for designing and synthesizing well-defined branched polymers and other complex architectures based on hyperbranched polymers [10]. Because self-condensing vinyl polymerization (SCVP) of inimers can produce hyperbranched polymers, it has been used to synthesize linear-dendritic hybrid block copolymers, multi-arm star polymers with hyperbranched cores, ‘pearl-necklace’ polymers with repeating hyperbranched pearls, and hypergrafted polymers with hyperbranched side chains on a linear polymer backbone [11–22]. However, most reported works involved SCVP of radical based inimers, such as (3-vinylphenyl)azomethylmalonodinitrile, and phenyl(4-vinylbenzyl)selane, showed low controllability of molecular weight [23–27]. Actually, only low molecular weight polymers (*M*_*w*_ < 10,000) were provided. To synthesize hyperbranched polymers with high molecular weight, copolymerizing inimers with styrene was implemented [28–30]. However, the copolymerizing strategy will definitely decrease the number of terminal functional groups. Therefore, the process of controlling the molecular weight of hyperbranched polymer while keeping the large number of terminal functional groups is a noteworthy challenge.

**Scheme 1.**
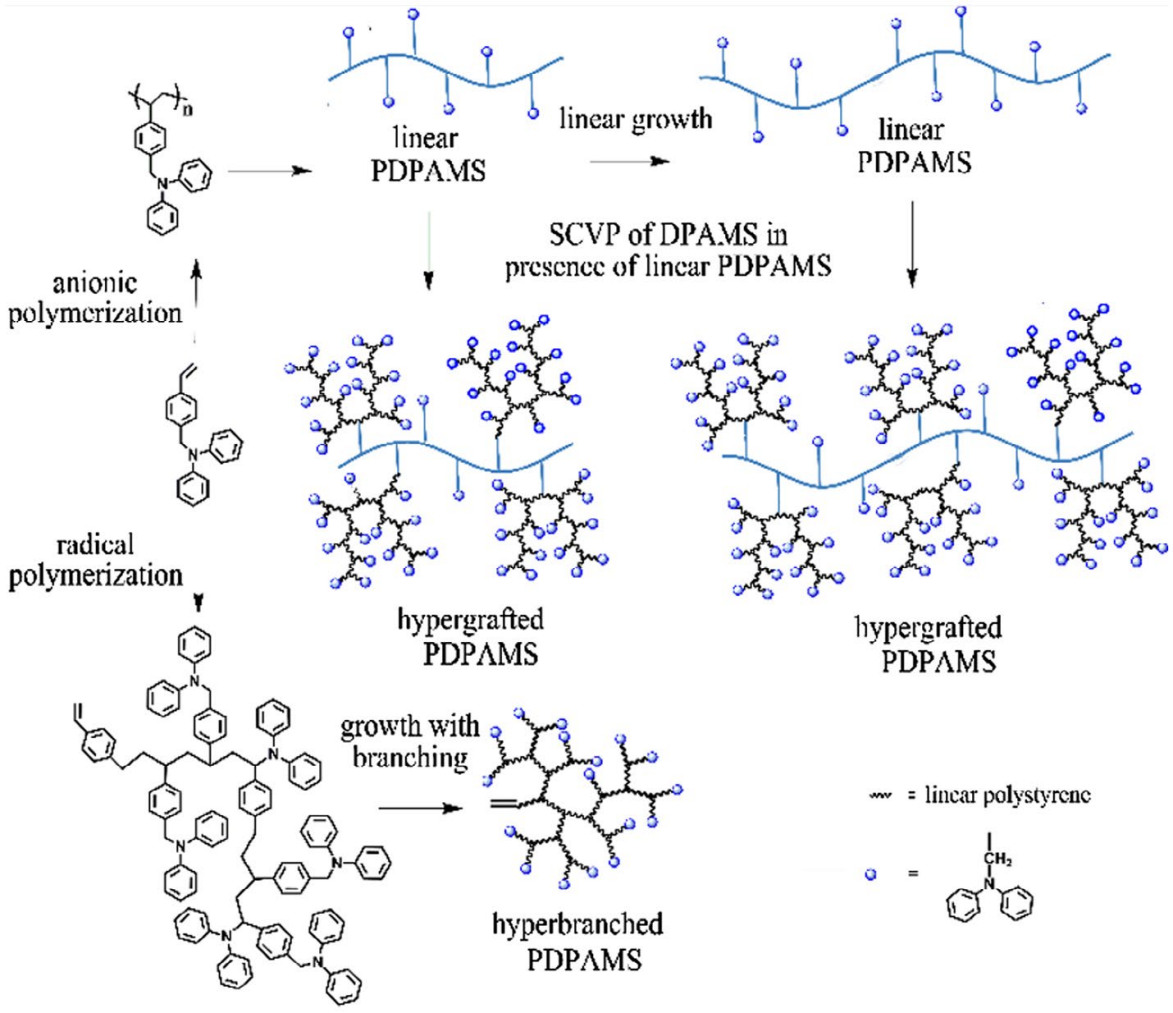
Synthesis of hyperbranched and hypergrafted PDPAMS.

To address this problem, we intend to graft hyperbranched polymers onto a linear backbone through tandem anionic polymerization and SCVP of a radical-inimer, which produce a hypergrafted polymer. Anionic polymerization is well-established as the best methodology for the synthesis of homopolymers, copolymers, and end-functionalized polymers with controlled polymer chain lengths and narrow molecular weight distributions [31–34]. We used this method to produce linear polymers with controlled chain length. The obtained polymers contained pendants of radical initiators, which were used to graft hyperbranched polymers obtained by SCVP of inimers to linear backbones. The mechanism of this approach is shown in Scheme 1. The goals of this work were to synthesize hypergrafted polymers with different molecular weights by controlling linear backbones, and to remain the number of terminal functional groups unchanged.

This paper discusses a new radical-based inimer, 4-(N,N-diphenylamino)methylstyrene (DPAMS), and the conditions for its anionic polymerization with a controlled linear polymer chain length. Hypergrafted PDPAMS was obtained using the SCVP of DPAMS in presence of linear PDPAMS. The results showed that the molecular weights of the hypergrafted polymers increased as the linear chain length increased. Thermal analysis showed that the hypergrafted polymers had lower glass transition and thermal degradation temperatures than hyperbranched and linear polymers did. Moreover, thermal crosslinkage of pendent N-benzyl-N-phenylaniline groups was observed in thermogravimetric analysis, resulting in multistep decomposition and a remaining weight of approximately 8–15 wt % at 700 °C.

## 2. Experimental section

### 2.1. Materials

All chemicals purchased from Innochem Company. Calcium hydride (CaH_2_), calcium chloride (CaCl_2_), ethanol, *n*-BuLi (2.5 M in cyclohexane), *sec*-BuLi (1.3 M in cyclohexane), *t*-BuLi (1.3 M in cyclohexane), 2,2,6,6-Tetramethylpiperidine 1-oxyland (TEMPO), diphenylamine, N-benzyl-N-phenylaniline (BDPA), and *p*-vinylbenzylchloride (*p*-VBC), were used as received. Toluene, Cyclohexane, and styrene (St) were refluxed over CaH_2_ and distilled under dry argon. Tetrahydrofuran (THF) was distilled from sodium naphthalene, before it was dried over Cacl_2_ for 48 h.

### 2.2. Characterization methods

^1^H spectra were recorded at 25 °C on a Brucker ARX400 (400 MHz) spectrometer with CD_2_Cl_2_ and tetramethylsilane as the solvent and internal reference, respectively. The apparent average molecular weights (*M*_*n*_, *M*_*w*_) and polydispersity index (PDI) of the polymers were determined with a refractive index size exclusion chromatography.

(RI-SEC; Waters SEC) equipped with a 515 high-performance liquid chromatography (HPLC) pump, 2410 refractive index detector, and three *μ*-Styragel columns (HT3 + HT4 + HT5). All samples were processed in THF at 30 °C at a rate of 1.0 mL/min. Linear PSt standards were used for calibration. The data were analyzed using a Waters Millennium 32 system. The absolute *M*_*w*_ and PDI of the polymers were determined with MALLS-SEC (Waters 1515 Isocratic HPLC Pump) equipped with DAWN EOS (Wyatt Technology) multiangle laser light scattering detector (MALLS) operating with a He–Ne laser (633 nm wavelength). The mass spectrum was conducted with a Waters ACQUITY ultra performance liquid chromatography coupled with quadrupole time-of-flight mass spectrometry (UPLC/Q-TOFMS) system using acetonitrile as solvent. Differential scanning calorimetry (DSC) was conducted with a Q2000 differential scanning calorimeter (TA Instruments, USA) under nitrogen purging at a heating rate of 10 °C min^−1^ from 0 to 160 °C. The thermogravimetric analysis (TGA) was conducted with Q5000-TA Instruments under nitrogen atmosphere from 30 to 750 °C with a heating rate of 10 °C/min.

### 2.3. Synthesis of 4-(N,N-diphenylamino)methylstyrene

**Scheme 2.**
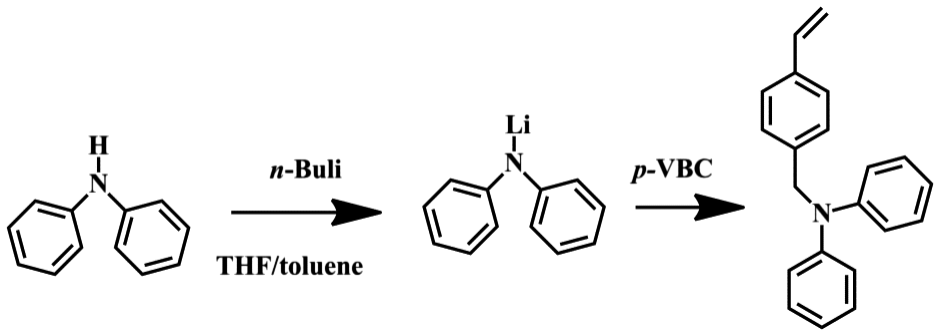
Synthesis of DPAMS.

As shown in Scheme 2, 4-(N,N-diphenylamino)methylstyrene (DPAMS) was prepared by the reaction of *p*-vinylbenzylchloride (*p*-VBC) and lithium diphenylamide, which is summarized as follows. Diphenylamine (6.8 g, 40.0 mmol) was dissolved in THF (100 mL) and then reacted with *n*-BuLi (16 mL, 40.0 mmol) at −78 °C for 6 h with the protection of nitrogen. Subsequently, *p*-VBC was added to the reactor to react with the lithium diphenylamide. The solution was stirred at 20 °C for 6 h and then poured into brine (100 mL). After extracted with diethylether and water, the reaction mixture was vacuum rotary evaporated to get the crude DPAMS. Then, the crude DPAMS was recrystallized thrice in ethanol to get faint yellow crystal.

### 2.4. Trapping radicals in the presence of TEMPO

DPAMS (0.28 g, 1 mmol) and TEMPO (0.31, 2 mmol) were mixed in toluene (1 mL) and reacted through stirring at 100 °C for 24 h under nitrogen. After the reaction, the resultant mixture was precipitated into excess methanol and then separated through filtration. The separated solution was characterized by UPCL/Q-TOF MS spectra.

### 2.5. Anionic polymerization of DPAMS

Anionic polymerization was implemented in a 50 mL glass reactor with the protection of nitrogen. First, glass reactors were flamed prior to use. DPAMS (2 g) was dissolved in 20 ml solvent (cyclohexane, toluene, or THF) with a 10 wt % solution in the flamed reactor. Subsequently, the required amount of BuLi (according to the [DPAMS]_0_/[BuLi]_0_ ratio summarized in Table 1) was added to the reactor from a hypodermic syringe to initiate the polymerization. The polymerization was stirred for 6 h and then terminated with methanol. The resultant polymers were precipitated into excess methanol to precipitate the polymer, which was then separated through filtration and dried in a vacuum oven to a constant weight.

**Table 1.** Effect of solvent, temperature and initiator on the anionic polymerization of DPAMS^a^.

	Solvent	Tem (°C)	Initiator	Yield (wt %)	*M*_*n*,design_^b^ (×10^−3^)	*M*_*n*,SEC_^c^ (×10^−3^)	PDI^c^
1	Cyclohexane	50	*n*-Buli	17.5	2.0	387.4	3.05
2	Toluene	50	*n*-Buli	61.3	2.0	63.1	2.81
3	THF	50	*n*-Buli	80.2	2.0	16.2	2.04
4	THF	30	*n*-Buli	78.5	2.0	8.3	1.73
5	THF	0	*n*-Buli	80.8	2.0	7.2	1.65
6	THF	−78	*n*-Buli	98.5	2.0	4.8	1.38
7	THF	−78	*t*-Buli	100	2.0	2.9	1.17
8	THF	−78	sec-Buli	100	2.0	2.6	1.14
9	THF	−78	sec-Buli	100	3.0	3.1	1.18
10	THF	−78	sec-Buli	100	5.0	4.8	1.16
11	THF	−78	sec-Buli	100	7.0	7.2	1.28
12	THF	−78	sec-Buli	100	12.0	14.2	1.32

^a^ Polymerization was carried out under a dry nitrogen atmosphere for 6 h.

^b^ *M*_*n*.design_: FW(C_21_H_19_N) × [DPAMS]_0_/[BuLi]_0_ × yield(%) × 10^−2^ + FW(C_4_H_9_).

^c^ *M*_*n*,SEC_ and PDI ^d^were estimated using a SEC by employing PSt as the standard.

### 2.6. Synthesis of hyperbranched PDPAMS

DPAMS (1 g) was added to toluene (2 mL). The mixture was stirred at room temperature for 10 min. The bottle was evacuated through three freeze–pump–thaw cycles, purged with purified argon, and then placed in a heated oil bath at 110 °C for 24 h. The resulting polymer was precipitated using ethanol, and then separated through filtration and dried in vacuum oven to a constant weight.

### 2.7. Synthesis of hypergrafted PDPAMS

Linear PDPAMS (0.1 g, obtained with anionic polymerization) and DPAMS (1 g) were added to toluene (2 mL). The mixture was stirred at room temperature for 10 min. The bottle was evacuated through three freeze–pump–thaw cycles, purged with purified argon, and then placed in a heated oil bath at 110 °C for 24 h. The resulting polymer was precipitated using ethanol, and then separated through filtration and dried in vacuum oven to a constant weight.

## 3. Results and discussion

### 3.1. Synthesis of DPAMS

**Figure 1.**
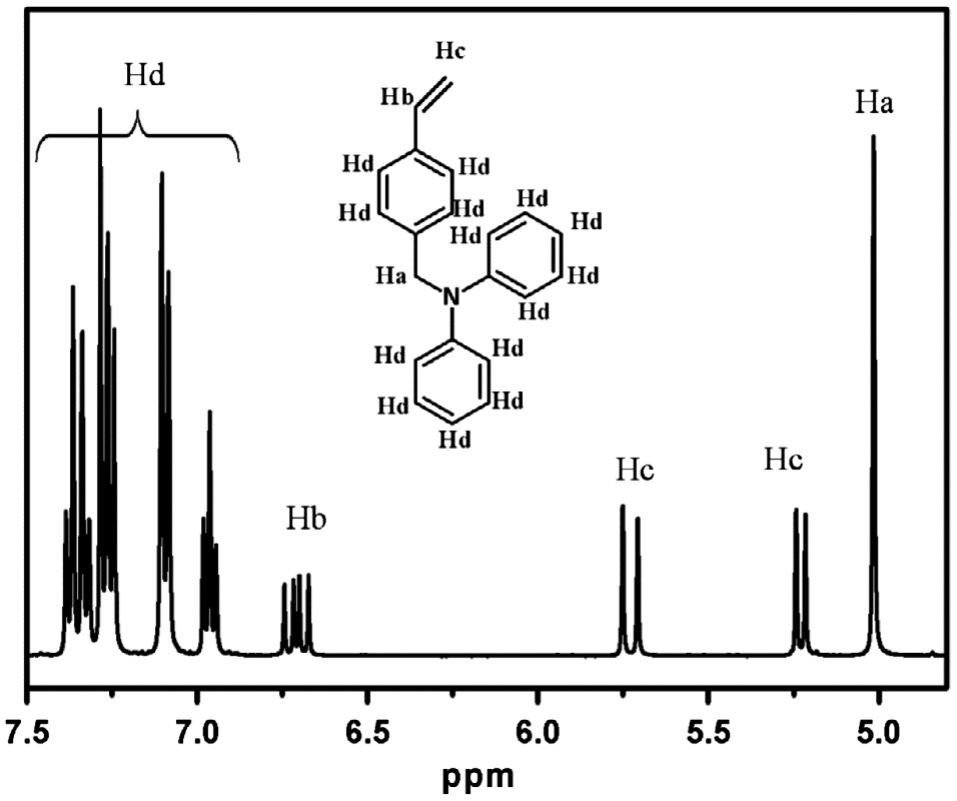
^1^H NMR spectrum of DPAMS.

In order to synthesis polymers with hypergrafted architecture, DPAMS that possesses a polymerizable vinyl group and initiator for radical polymerization was prepared by the reaction of *p*-vinylbenzylchloride (*p*-VBC) and lithium diphenylamide (Scheme 2). Successful synthesis of this functionalized inimer was confirmed by ^1^H NMR spectra (Figure 1). The peaks from 6.0 to 7.3 ppm are assigned to aromatic protons in the DPAMS. The peaks from at approximately 6.7 ppm, 5.7, and 5.2 are assigned to vinyl protons. The peak at approximately 5.0 ppm is assigned methylene protons adjacent to nitrogen atom. The proton rations correlate well with the formula of DPAMS.

**Figure 2.**
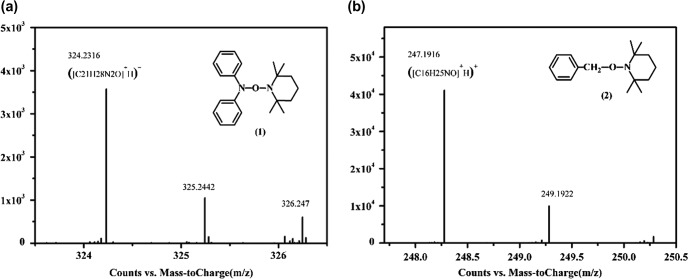
TOF MS (ES+) spectrum of structure trapped by TEMPO.

**Figure 3.**
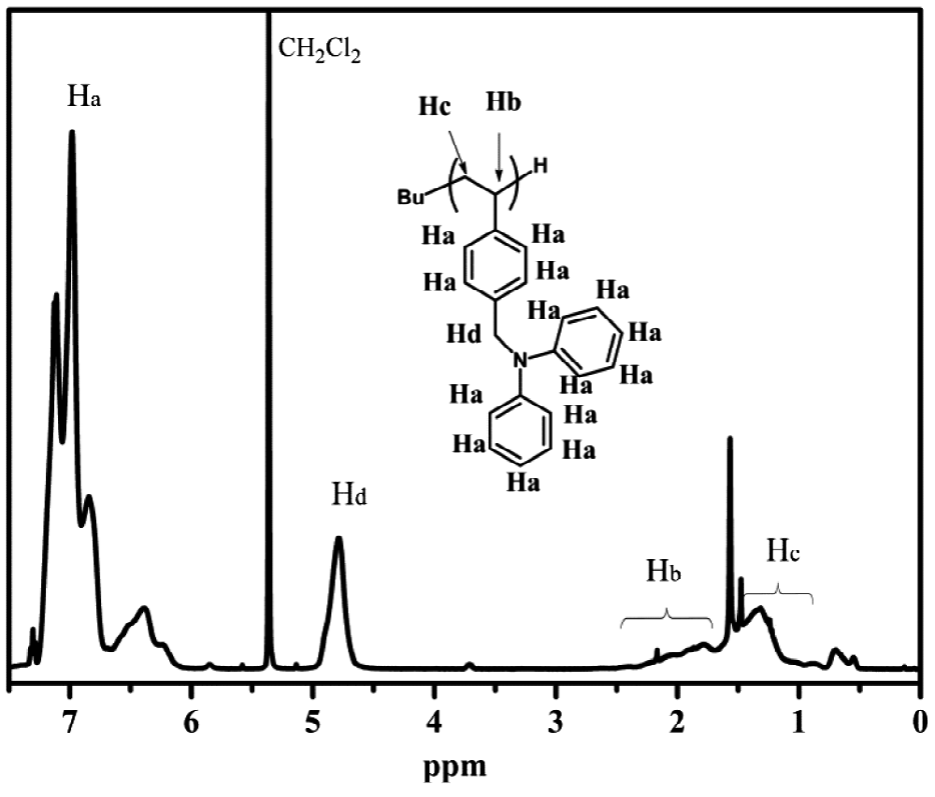
^1^H NMR spectrum of linear PDPAMS synthesis through anionic polymerization.

**Figure 4.**
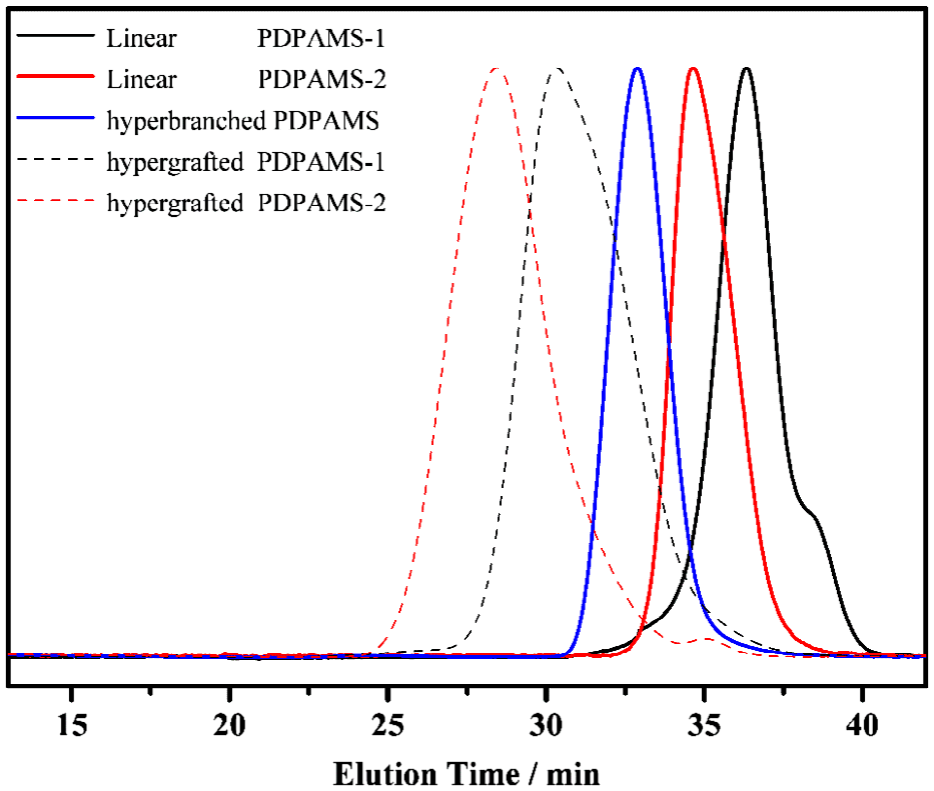
SEC profiles of linear, hyperbranched, and hypergrafted PDPAMS.

**Figure 5.**
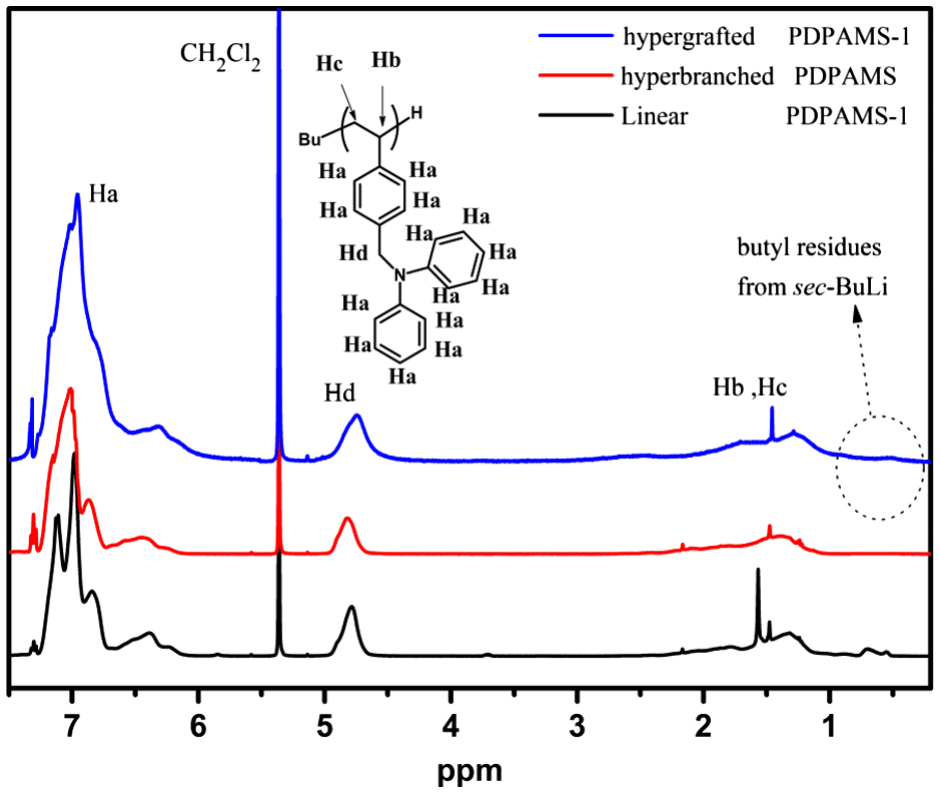
^1^H NMR spectra of linear, hyperbranched, and hypergrafted PDPAMS.

**Figure 6.**
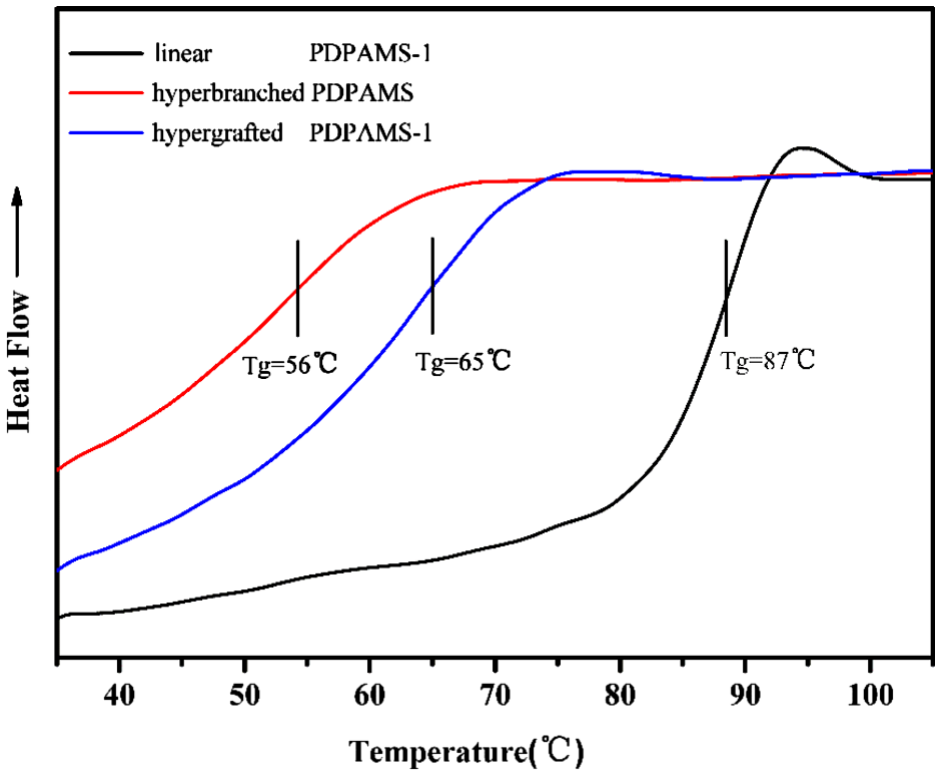
DSC curves of Linear, hyperbranched, and hypergrafted PDPAMS.

**Figure 7.**
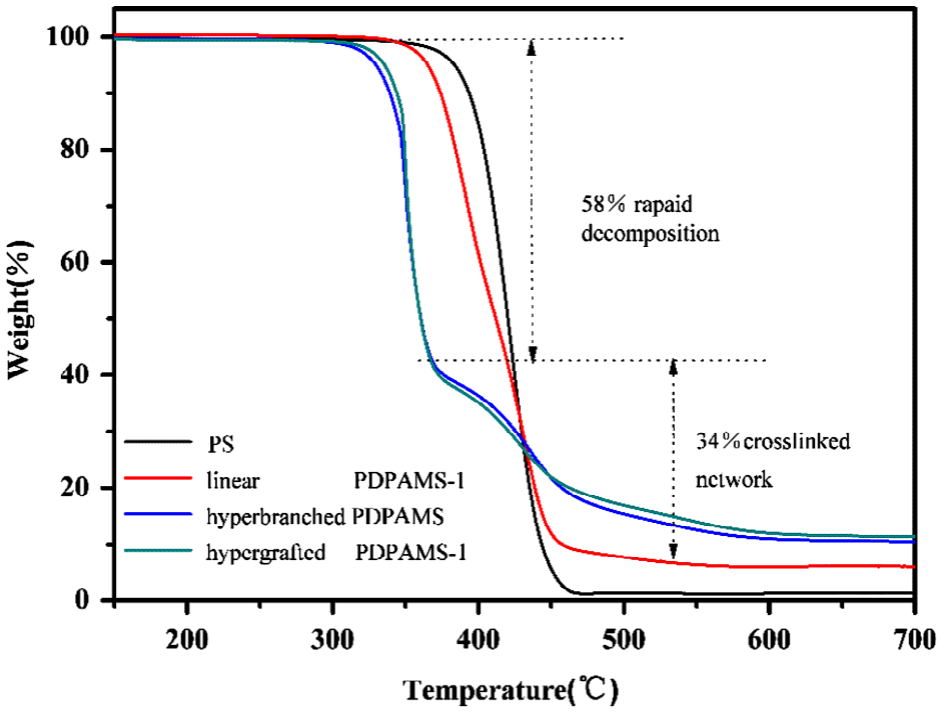
TGA curves of PS, Linear, hyperbranched, and hypergrafted PDPAMS.

**Scheme 3.**
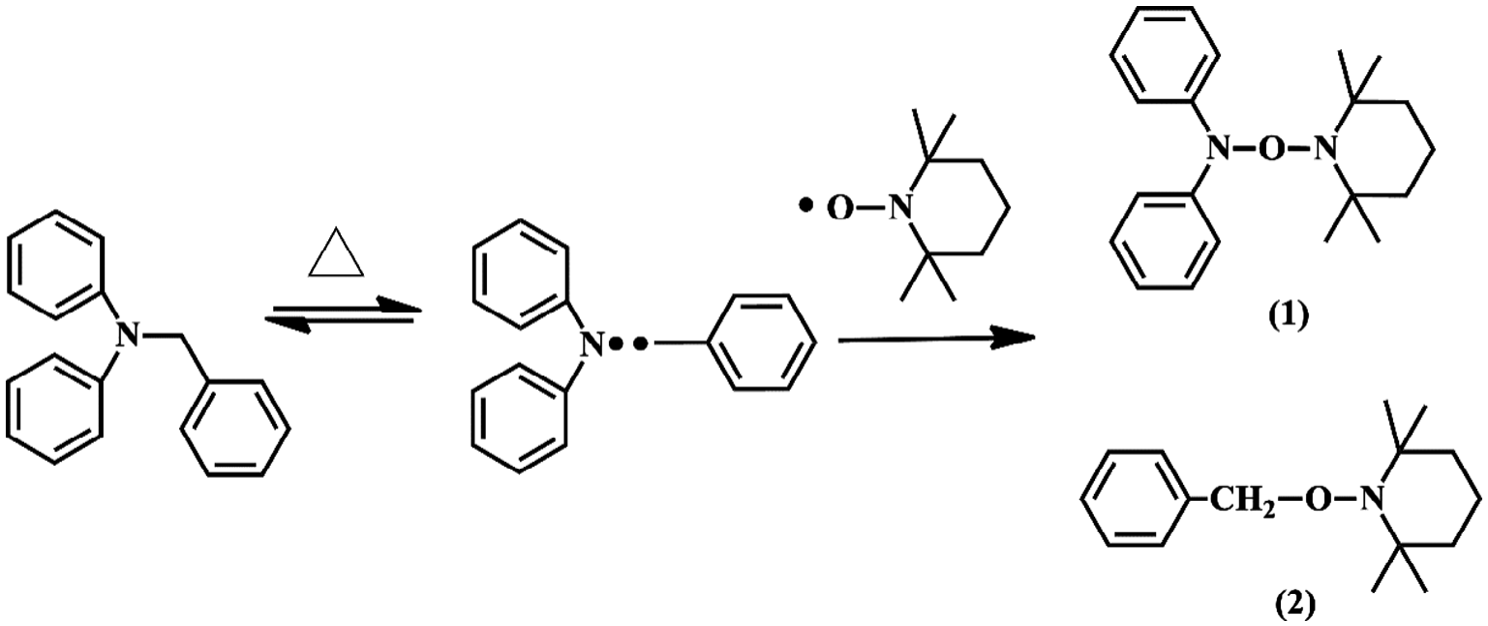
Possible dissociation mechanism of BDPA.

To prove that DPAMS was a typical of radical-based inimer, we initially attempted to study the dissociation of BDPA on the side chain of DPAMS. TEMPO was used as radical trapper to trap the radicals dissociated by BDPA (Scheme 3). Figure 2 shows the TOF MS (ES+) spectra of structure trapped by TEMPO. In Figure 2 (1), the TOF MS (electrospray ionization) m/z [M + H] ^+^ were 324.231, 325.244, and 326.247 showed good agreement with molecular weight calcd for C_21_H_28_N_2_O. Figure 2 (2), the TOF MS (electrospray ionization) m/z were 247.1916 and 249.1922 showed good agreement with molecular weight calcd for C_16_H_25_NO. These results confirm the existence of diphenylamide radicals in Scheme 3. Therefore, we can conclude that thermolysis of BDPA results in an initiating benzyl radical and an inactive diphenylamide radical. In addition, the BDPA group on the side chain of DPAMS can serve as a radical initiator (proved in Figs. S1–S4). On the basis of these results, we conclude that DPAMS was a typical of radical-based inimer.

### 3.2. Synthesis of linear PDPAMS via anionic polymerization

**Scheme 4.**
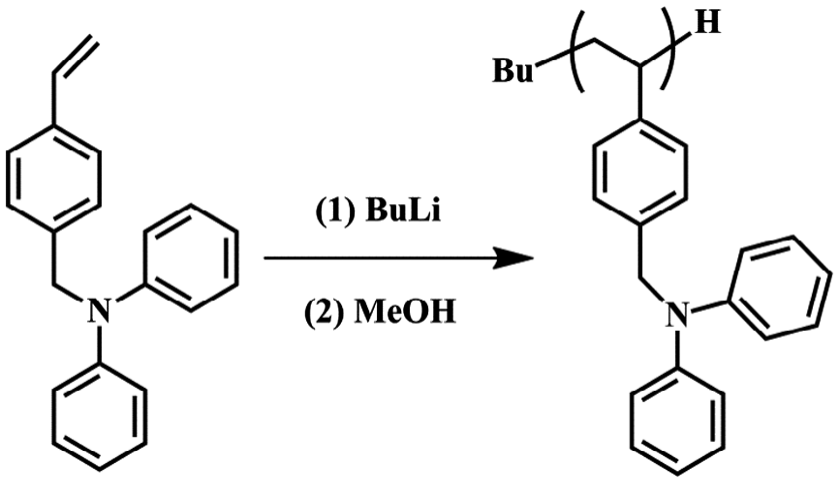
Anionic polymerization of DPAMS with *n*-BuLi in THF at −78 °C.

As shown in Scheme 1, the combination of living anionic polymerization and SCVP using DPAMS as an inimer is based on a two-step strategy. In the first step, a linear backbone that had well-controlled polymer chain lengths and incorporated latent radical initiating sites was synthesized through anionic polymerization of DPAMS. The obtained linear PDPAMS was subsequently used as a ‘hyperlinker’ to link hyperbranched side chains, which were formed through SCVP of DPAMS. As a results, the tandem anionic polymerization and SCVP of DPAMS gave rise to a hypergrafted PDPAMS. However, DPAMS was substituted with a diphenylamide in the para position of the phenyl ring (Scheme 4). Therefore, it was believed that the anionic polymerization of DPAMS was difficult to control because of the strong nucleophilicity of the nitrogen atoms in DPAMS [34–36]. The anionic polymerization of DPAMS was carried out under different conditions, and the results obtained from these polymerization processes are displayed in Table 1.

When cyclohexane and toluene were used as solvents at 50 °C, PDPAMS precipitated in the corresponding solvent as red compounds, resulting in a lower yield and a large PDI value (Table 1, nos. 1 and 2). When THF was used as a solvent, the yield was high because of the increased solubility of DPAMS and PDPAMS (Table 1, no. 3). Therefore, THF is a more favorable solvent for the anionic polymerization of DPAMS. However, a large PDI value was still observed under this condition, because BuLi and THF tend to form a monomeric complex in a solution, and this complex tends to causes the metalation reaction of organic compounds with acidic hydrogens [37,38]. Therefore, the initiator and propagating carbanions might be deactivated by methyl groups adjacent to the amino atom, and a low temperature should be implemented to avoid these unfavorable side reactions. Unsurprisingly, the PDI value decreased with decreasing temperature (Table 1, nos. 3–6). When the PDI value was low and the *M*_*n*,SEC_ showed close agreement to *M*_*n*,design_ at −78 °C with *sec*-BuLi as the initiator, indicating that side reactions were notably suppressed under this condition. Subsequently, the anionic polymerization of DPAMS was implemented with different [DPAMS]/[*sec*-BuLi] molar ratios to examine the characteristics of this polymerization condition (Table 1, nos. 8–12). All of the PDPAMS obtained showed low PDI and quantitative yield. With increasing [DPAMS]/[*sec*-BuLi] molar ratios, the *M*_*n*_ of PDPAMS increased and showed close agreement with *M*_*n*_ design from [DPAMS]/[*sec*-BuLi] molar ratios, indicating the highly controlled nature of these polymerizations. Therefore, linear PDPAMS with different chain lengths can be obtained through anionic polymerization.

Figure 3 shows the ^1^H spectrum of PDPAMS obtained through anionic polymerization (Table 2, no. 8). The peaks ranging from 6.0 to 7.26 (*H*_*a*_) ppm can be assigned to aromatic protons in DPAMS and St. The peak at approximately 4.9 (*H*_*d*_) was contributed by methylene protons adjacent to nitrogen atoms in DPAMS. The peaks from 1.2 to 2.9 (*H*_*b*_, *H*_*c*_) ppm can be assigned to methylidyne and methylene protons in the main chain. The peaks from 0.5 to 0.9 ppm were the characteristic peaks of *sec*-BuLi. The ratio of *H*_*d*_/*H*_*b*_ + *H*_*c*_/*H*_*a*_ was 2/3/14. The proton ratio strongly supported the polymer chain structure for DPAMS that is shown in Figure 1. On the basis of these results, we concluded that DPAMS was successfully initiated by *sec*-BuLi with linear chains.

**Table 2.** Molecular weights and polydispersity indexes values for linear, hyperbranched, and hypergrafted PDPAMS.

No.	Samples	RI-SEC		MALLS-SEC	
		*M*_*w*_ × 10^-4^	PDI	*M*_*w*_ × 10^-4^	PDI
1	Hyperbranched PDPAMS	1.78	1.98	5.21	2.32
2	Linear PDPAMS-1	0.92	1.35	0.76	1.24
3	Linear PDPAMS-2	1.98	1.16	2.03	1.05
4	Hypergrafted PDPAMS-1	3.05	1.87	6.53	1.91
5	Hypergrafted PDPAMS-2	8.07	3.77	15.94	3.36

### 3.3. Synthesis of hyperbranched and hypergrafted PDPAMS

It was proved that DPAMS was a radical-based inimer. As shown in Scheme 1, the benzyl radical can react with vinyl group of a second monomer of DPAMS to produce dimer. Repeating this process, the hyperbranched PDPAMS will be formed. The structure of hyperbranched PDPAMS was confirmed by Multiangle laser light scattering (MALLS) measurements. As shown in Table 2, nos. 1, The values of *M*_*w*_ determined by MALLS-SEC was notably higher than the corresponding *M*_*w*_ values determined using RI-SEC for hyperbranched PDPAMS, indicating that branched structures may exist in the samples we synthesized [13,39–42]. This happened because branched polymers have smaller hydrodynamic volumes than a linear polymer [23,40]. However, the molecular weight did not satisfactory increase even after the prolonged reaction period (Fig. S5). This result was in consistent with the SCVP of radical based inimers [14,23,27].

It was proved in Figs. S6–S9 that hypergrafted PDPAMS can be prepared by heating DPAMS with linear PDPAMS in toluene at 110 °C for 24 h. In this work, we employed this polymerization with two different linear PDPAMS (linear-1 and linear-2 obtained by anionic polymerization). To confirm the hypergrafted structure, the linear PDPAMSs and hypergrafted PDPAMSs obtained were also characterized by conventional RI-SEC and MALLS-SEC. The results are shown in Table 2 and Figure 4. For linear PDPAMS the values of *M*_*w*_ determined by MALLS-SEC were approximately equal with the *M*_*w*_ values determined using RI-SEC (Table 2, nos. 2, 3). In contrast, the values of *M*_*w*_ determined by MALLS-SEC were notably higher than the corresponding *M*_*w*_ values determined using RI-SEC for hypergrafted PDPAMS, indicating that branched structures may exist in the samples we synthesized (Table 2, nos. 4, 5).

As shown in Figure 4, the molecular weight of hypergrafted PDPAMSs were higher than hyperbranched and linear PDPAMS. Considering that the molecular weight of hyperbranched PDPAMS unchanged after prolonged reaction period (Fig. S5), these increases of hypergrafted PDPAMS molecular weights were ascribed to the addition of linear PDPAMS. We speculated that the benzyl radical on the side chain of linear PDPAMS reacted with the carbon-carbon double bond of DPAMS, which followed by self-condensing vinyl polymerization of DPAMS, or coupled with hyperbranched PDPAMS free radical, leading the increased molecular weight. Moreover, with the increasing length of linear PDPAMS, the molecular weight of hypergrafted PDPAMS was also increased from hypergrafted PDPAMS-1 to hypergrafted PDPAMS-2 (Figure 4). This increase of molecular weight indicates that more hyperbranched fragment will be linked onto linear PDPAMS with a longer length. Although the hypergrafted PDPAMSs obtained by this strategy were mixed with a proportion of hyperbranched PDPAMS, SiO_2_ particles could be used to separate hyperbranched PDPAMS (Figs. S6–S9). We should point out that the part of hypergrafted PDPAMS and hyperbranched PDPAMS overlapped in SEC profile was small, indicating the proportion of hyperbranched PDPAMS in hypergrafted PDPAMS was very low. Therefore, we concluded that this strategy can be implemented to control the molecular weight of hypergrafted PDPAMS by changing the length of linear backbone.

The ^1^H spectra were measured to evaluate the structures of linear, hyperbranched, and hypergrafted PDPAMS. As shown in Figure 5, the characteristic peaks of PDPAMS appeared in every ^1^H spectrum. In addition, the proton ratio of *H*_*a*_/*H*_*d*_ in linear PDPAMS was 7, whereas this proton ratio in hyperbranched PDPAMS was 7.9, indicating the existence of hyperbranched structures in hyperbranched PDPAMS. The increased proton ratio was mainly attributed to the initiation of N-benzyl-N-phenylaniline pendants in DPAMS, which reduced the number of *H*_*d*_ protons. The *H*_*a*_/*H*_*d*_ proton ratio was 9.9 in hypergrafted PDPAMS, indicating higher branching density. The ^1^H NMR spectra suggested that the degree of branching for hypergrafted PDPAMS is higher than hyperbranched PDPAMS. Meanwhile, the peaks of sec-BuLi ranging from 0.5 to 0.9 ppm appeared in the spectra of both linear and hypergrafted PDPAMS. Considering that the SEC profile of hypergrafted PDPAMS, linear PDPAMS and hyperbranched PDPAMS overlapped was small, we speculated that hyperbranched PDPAMS was successfully grafted onto the backbone of linear PDPAMS. On the basis of these results, we concluded that hypergrafted PDPAMS was successfully synthesized by combining anionic polymerization and SCVP of DPAMS.

### 3.4. Thermal analysis

Polymer structures exhibit notable differences in glass transition temperatures and thermal stability levels. Therefore**,** the thermal behaviors of linear, hyperbranched, and hypergrafted PDPAMS were characterized by DSC and TGA (Figures 6 and 7). DSC curves of PDPAMS as shown in Figure 6, hyperbranched and hypergrafted PDPAMS have a lower *T*_*g*_ than linear PDPAMS does, because the structures of hyperbranched polymers and hypergrafted polymers have more chain ends and their segments are more mobile. Therefore, hyperbranched and hypergrafted PDPAMS have larger free volumes and lower *T*_*g*_ values [17,43]. On the basis of these results, we conclude that branched structures existed in the samples of hyperbranched and hypergrafted PDPAMS.

Figure 7 shows the TGA curves of PS, and of linear, hyperbranched, and hypergrafted PDPAMS. In contrast to PS, which exhibited only one distinct step of decomposition, all the PDPAMS samples showed two distinct steps of decomposition. The first decomposition step occurred from 330 to 410 °C and then leveled off at temperatures higher than 500 °C with remaining weights of approximately 8–15 wt %. The first decomposition step may have resulted from the evaporation of diphenylamide at the benzylic position. PDPAMS contained abundant quantities of pendent BDPA and the bond energy of the C–N bond was lower than that of the C–C bond. At the first temperature range, BDPA dissociated at the benzylic position of PDPAMS, and the residual benzylic moiety underwent crosslinkage to form a network structure, leading to a final product, the remaining weight of which was measured at 700 °C. The PDPAMS lost weight; the weight loss at 700 °C was greater than the weight loss at 410 °C, which was mainly attributed to the chain scission of the PDPAMS backbone. In addition, both the hyperbranched and hypergrafted PDPAMS showed lower stability than linear PDPAMS did, and both had lower initial decomposition onset temperatures. This phenomenon can be attributed to the hyperbranched benzylic N-benzyl-N-phenylaniline structure, which is relatively prone to thermal degradation.

## 4. Conclusion

In conclusion, a new radical-based inimer DPAMS was synthesized and the living controlled anionic polymerization of DPAMS was performed in THF at −78 °C using *sec*-BuLi as the initiator; Hyperbranched PDPAMS was synthesized through SCVP of DPAMS; Hypergrafted PDPAMS was synthesized through SCVP of DPAMS in the presence of linear PDPAMS. The hyperbranched and hypergrafted structure was confirmed by the comparison of RI-SEC and MALLS-SEC, ^1^H spectrum, DSC, and TGA analysis. Meanwhile, the molecular weights of hyperbranched PDPAMS remain unchanged even though prolong the reaction time. While, the molecular weights of hypergrafted polymers were controlled by changing the linear backbone chain length. Although the hypergrafted PDPAMSs were mixed with a proportion of hyperbranched PDPAMS ungrafted onto the linear PDPAMS, our work gives a new strategy controlling the molecular weight of hypergrafted PDPAMS.

## Disclosure statement

No potential conflict of interest was reported by the authors.

## Funding

This work was supported by National Natural Science Foundation of China [grant number 51473010] and National Basic Research grogram of China [grant number 2015CB654701].

## Supplemental data

The supplemental data for this article is available online at https://doi.org/10.1080/15685551.2017.1365577.

## Supplementary Material

TDMP_1365577_Supplementary_Material.docx
