# Supplementary material for: Synthesis of hypergrafted poly[4-(N,N-diphenylamino)methylstyrene] through tandem anionic-radical polymerization of radical-inimer
Source: Des Monomers Polym. 2017 Aug 30;20(1):476–84. doi: 10.1080/15685551.2017.1365577 (PMC5784871; doi:10.1080/15685551.2017.1365577)
Supplement: TDMP_1365577_Supplementary_Material.docx [file TDMP_A_1365577_SM4122.docx]

Supporting Information：

**Synthesis of hypergrafted poly[4-(N,N-diphenylamino)methylstyrene] through tandem anionic-radical polymerization of radical-inimer**

**Minglu Huang, Jianmin Lu*,Bingyong Han*, Xianhong Zhang, Wantai Yang**

*State Key Laboratory of Chemical Resource Engineering,* *Beijing University of Chemical Technology, Beijing, 100029, P. R. China.*

*****Correspondence authors

Email: [lujm@mail.buct.edu.cn](mailto:lujm@mail.buct.edu.cn); [hanby@mail.buct.edu.cn](mailto:hanby@mail.buct.edu.cn)

**S1. Possible mechanism for the polymerization of St initiated by BDPA**





**S2. Time-conversion relationship for the polymerization of St in presence of BDPA**





**S3. SEC profile for the polymerization of St in presence of BDPA**





**S4. ^1^H spectra of PS obtained by the polymerization of St in presence of BDPA**





DPAMS contained a polymerizable double bond and a pendent of BDPA. To demonstrate that DPAMS was a radical-based inimer, we should firstly prove BDPA was a thermal radical initiator. Hence, the radical polymerization of St was performed using BDPA as an initiator in toluene at 100 °C and a comparative experiment was implemented without BDPA (Fig. S1). BDPA (0.1 g) and St (1mL) were added to toluene (2 mL). As a comparative experiment, St (1mL) was added to toluene (2 mL) without BDPA. The mixtures were stirred at room temperature for 10 min. The bottle was evacuated through three freeze–pump–thaw cycles, purged with purified nitrogen, and then placed in a heated oil bath at 100 °C for 24 h. The resulting polymer was precipitated using ethanol and dried in vacuum oven to constant weight. No polymer obtained in the experiment without BDPA, indicating St was initiated by BDPA. As shown in Fig. S2 and Fig. S3, the monomer conversion and M_n_ increased progressively with time, indicating that this polymerization shows a relatively living nature in limited conversion ranges. It is well known that molecular weights of polymers obtained by conventional radical polymerization are unchanged during polymerization. Fig. S4 showed the typical ^1^H spectra of PS obtained by the polymerization. The peaks from 6.0-7.2 ppm were aromatic protons in St. Based on the above result, we conclude that St was successfully initiated by BDPA and therefore DPAMS was proved to be a typical of radical-based inimer.

**S5. SEC profiles of hyperbranched PDPAMS obtained by SCVP in different polymerization time**





**S6. synthetic routes to linear and hypergrafted PDPAMS grafted SiO_2._**





**S7. Fourier transform infrared spectrograms of SiO2, linear PDPAMS grafted SiO2, and hypergrafted PDPAMS grafted SiO2.**





**S8. Thermogravimetric analysiscurves of SiO2, linear PDPAMS grafted SiO2, and hypergrafted PDPAMS grafted SiO2.**





**S9. SEC profiles of linear, hyperbranched, and hypergrafted PDPAMS**





In order to prove that hyperbranched PDPAMS can be grafted onto linear PDPAMS, core-shell hybrid particles with inner solid SiO2 core surrounded by linear and hypergrafted PDPAMS was synthesized. As shown in Fig. S6, the core-shell hybrid particles were prepared by the combination of sequential anionic polymerization of DPAMS, following the radical polymerization of DPAMS. In the first step, Linear PDPAMSLi (2g, 0.25 mmol, obtained with anionic polymerization) was terminated with silicon tetrachloride (0.5 mmol) in cyclohexane at room temperature. This process allowed end-capping the PDPAMS living chains with chlorine (PDPAMScl). Then SiO_2_ (1g) was added to the reaction solution to obtain SiO_2_ linear PDPAMS hybrid particle. The resulting particle was precipitated using ethanol, and then separated through filtration. The separated SiO_2_ particles was washed with THF several times to remove the ungrafted PDPAMS until no remaining precipitated PDPAMS was detected in the supernatant solution by using the excess methanol. The particles were dried in a vacuum at 60 °C. In the second step, SiO_2_ linear PDPAMS hybrid particles (1.2 g) and DPAMS (1 g) were added to toluene (2 mL). The mixture was stirred at room temperature for 10 min. The bottle was evacuated through three freeze–pump–thaw cycles, purged with purified argon, and then placed in a heated oil bath at 110 °C for 24 h. The resulting particles were precipitated using ethanol. The separated SiO2 particles was washed with THF several times to remove the ungrafted PDPAMS until no remaining precipitated PDPAMS was detected in the supernatant solution by using the excess methanol. The ungrafted PDPAMS was produced by SCVP of DPAMS and therefore was a hyperbranched PDPAMS. In addition, the existence of hyperbranched PDPAMS demonstrated some hyperbranched PDPAMS will remain in the hypergrafted PDPAMS without proper separation process. The particles were dried in a vacuum at 60 °C. Then, all SiO_2_ hybrid particles were placed into an aqueous hydrofluoric acid (HF) solution with 5 vol % HF (a mixed solution of hydrofluoric acid (HF, 10 ml) with ultrapure water (70 ml)) to perform the cleavage of grafted PDPSMS.

Fig. S7 shows the Fourier transform infrared spectrograms (FTIR) of SiO2, linear PDPAMS grafted SiO2, and hypergrafted PDPAMS grafted SiO2. The characteristics of linear and hypergrafted PDPAMS grafted SiO2 particles were notably different from those of SiO2, namely in the presence of new peaks at 2965, 2920, 2852, 1510, 1438, and 712 cm−1. The absorptions at 2965, 2920 and 2852 cm−1 shown on the spectra of PDPAMS grafted SiO2 are attributed to the stretching vibration of the methylene bond. The peaks at 1510 and 1438, and 701 were assigned to benzene ring absorption of PDPAMS. The FTIR spectra indicated that the PDPAMS successfully grafted onto the surface of SiO2.

TGA was conducted to evaluate the grafting content of PDPAMS. As shown in Fig. S8, pure SiO2 exhibited approximately 2.5 % weight loss between 50 °C and 800 °C. The weight loss of linear and hypergrafted PDPAMS grafted SiO2 particles was 6.5 % and 12.7 %, respectively. The weight loss difference between PDPAMS grafted SiO2 particles and pure SiO2 was measured as the content of the PDPAMS grafted onto the SiO2. The PDPAMS weight content in the linear and hypergrafted PDPAMS grafted SiO2 particles was approximately 4% and 10.2%, respectively. The increased PDPAMS weight content was attributed to the linkage of hyperbranched PDPAMS to linear PDPAMS.

Fig. S9 shows the SEC profiles of linear, hyperbranched, and hypergrafted PDPAMS. For linear PDPAMS, the SEC profile was bimodal, indicating some coupled PDPAMS in the resulting linear PDPAMS. These coupled PDPAMS resulted from linking PDPAMSLi anions with silicon tetrachloride. The SEC profile of the hypergrafted PDPAMS clearly shifted toward the higher molecular weight region more than that of the linear and hyperbranched. Meanwhile, the molecular weight of hyperbranched PDPAMS unchanged after prolonged reaction period (Fig. S5). Therefore, we speculated that hyperbranched PDPAMS was linked with linear PDPAMS by heating DPAMS with linear PDPAMS.

Based on the above result, we conclude that hyperbranched PDPAMS can be grafted onto linear PDPAMS and therefore a hypergrafted PDPAMS was synthesized.
